# Uncovering the Atomic Structure of Substitutional Platinum Dopants in MoS_2_ with Single-Sideband Ptychography

**DOI:** 10.1021/acs.nanolett.5c00919

**Published:** 2025-05-23

**Authors:** David Lamprecht, Anna Benzer, Manuel Längle, Mate Capin, Clemens Mangler, Toma Susi, Lado Filipovic, Jani Kotakoski

**Affiliations:** † 27259 Institute for Microelectronics, TU Wien, Gußhausstraße 25-29, 1040 Vienna, Austria; ‡ Faculty of Physics, University of Vienna, Boltzmanngasse 5, 1090 Vienna, Austria

**Keywords:** 4D-STEM, Ptychography, MoS_2_, Pt dopants, 2D materials, SSB

## Abstract

We substitute individual Pt atoms into monolayer MoS_2_ and study the resulting atomic structures with single-sideband ptychography (SSB) supported by *ab initio* simulations. We demonstrate that while high-angle annular dark-field (HAADF) scanning transmission electron microscopy (STEM) imaging provides excellent *Z*-contrast, distinguishing some defect types such as single and double sulfur vacancies remains challenging due to their low relative contrast difference. However, SSB with its nearly linear *Z*-contrast and high phase sensitivity enables reliable identification of these defect configurations, as well as various Pt dopant structures at significantly lower electron doses. Our findings uncover the precise atomic placement and highlight the potential of SSB for detailed structural analysis of dopant-modified 2D materials while minimizing beam-induced damage, offering new pathways for understanding and engineering atomic-scale features in 2D systems.

## Graphical abstract

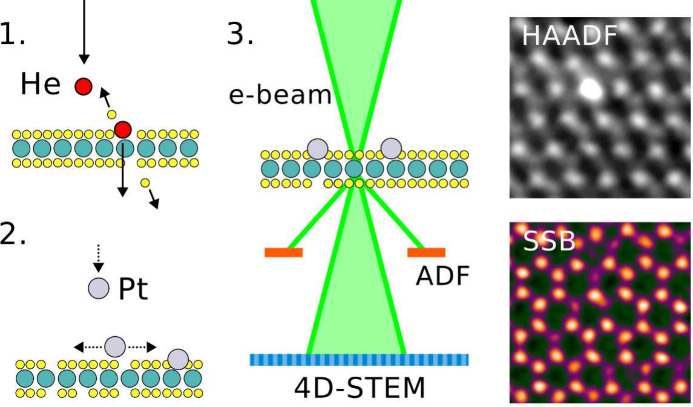

Due to their high surface-to-volume ratio, 2D materials are promising candidates as active materials for future catalytic and gas-sensing applications. Particularly MoS_2_, which as a monolayer is an intrinsic direct band gap semiconductor, has attracted significant interest. However, a major limitation of MoS_2_ as a catalytic material is the relative chemical inertness of its basal plane, which severely restricts its potential use cases.^1^ To overcome this problem, various material modification methods like surface metal decoration,^2,3^ defect-engineering,^4,5^ or the assembling of heterostructures with other 2D materials^6,7^ have been proposed and experimentally verified. Substitutional doping, where a single heteroatom replaces one or more atoms in the lattice, is considered a modification method of particular interest^8^ due to its simplicity and potentially high selectivity. Replacement of S atoms has been reported for over half of the elements on the periodic table,^9^ but atomic resolution confirmation of this incorporation remains scarce.

Substitution to chalcogen sites has been achieved either with direct incorporation during chemical vapor deposition (CVD) growth, e.g., O,^10^ Va,^11^ alloying with other chalcogens, e.g., Se,^12^ post-growth plasma implantation, e.g., N,^13^ Cl,^14^ or various hydrothermal methods, e.g., Rh,^15^ W.^16^ Evidence for the substitution of Mo atoms is limited to bottom-up methods and mostly performed by adding metals to the precursor during CVD growth, e.g., Fe,^17^ Ta,^18^ and hydrothermal growth methods, e.g., Pd.^19^ Notably, there are few reports of implanting precious metals like Pt into MoS_2_, despite various theoretical predictions regarding the potential of Pt-doped MoS_2_ for gas-sensing and catalysis.^20–22^

In ref 23., Li et al. studied single Pt atoms on MoS_2_ by separating individual atoms from clusters using the electron beam of an aberration-corrected scanning transmission electron microscope (STEM). They were able to successfully implant single Pt atoms into S vacancy sites and study their dynamics under an electron beam. However, this substitution method is barely scalable and is not trivially adaptable to other elements or atomic sites. Several other studies have claimed selective substitution of Mo atoms with Pt^21,24,25^ atoms, but none have provided atomic-resolution confirmation, which is essential to distinguish between true lattice incorporation and mere surface decoration.

In this study, we extend our previously published two-step implantation method, originally demonstrated for implanting graphene with Au,^26,27^ Fe, Ag, Ti,^26^ and Al,^26,28^ to MoS_2_. First, we introduce defects into monolayer MoS_2_ using low-energy He ion irradiation with a plasma source.^29^ Subsequently we fill the vacancies with single Pt atoms stemming from an evaporation source. For structural analysis of the modified material, high-angle annular dark-field (HAADF)-STEM and simultaneous 4D-STEM imaging are carried out. The resulting 4D data stacks are used to reconstruct the phase information using the single-sideband ptychography (SSB) algorithm.^30^

While in HAADF imaging the intensity of an individual atom scales with the atomic number^31^ typically as *Z* ^1.64^, the phase contrast in SSB is approximately linear to the amplitude of the projected potential^32^ *Z*, which results in an approximately linear dependence on *Z* for single atoms. This allows the simultaneous and precise imaging of neighboring heavy and light atoms with SSB, which is needed for the analysis of our structures.^33–35^ We show that SSB allows us to reliably differentiate not only between different vacancy structures but also between Pt atoms trapped in single (V_1S_) and double S vacancies (V_2S_), a distinction that is challenging in HAADF-STEM due to their low contrast difference. To obtain further evidence for the correct assessment of the defect structures, we compare the obtained experimental images with image simulations and study the vacancy-mediated substitution process using density functional theory (DFT). Overall, as our method relies on the filling of vacancies by adatoms, our results demonstrate a pathway for the controlled substitutional doping of MoS_2_ with arbitrary elements.

CVD-grown MoS_2_ samples were transferred from the SiO_2_ substrate to Quantifoil Au TEM grids and subsequently introduced into a interconnected UHV system.^36^ The sample contains large atomically clean areas with a low density of intrinsic defects when investigated with STEM. After initial imaging, the samples were transferred in UHV to the sample manipulation chamber and exposed to He ions with a kinetic energy of 171 ± 21 eV, which is the intrinsic kinetic energy for He ions of the used plasma source at a He partial gas pressure of ca. 2.5 × 10^–5^ mbar. The sample treatment is illustrated in Figure 1, including atomic-resolution images at the different stages. To produce a defect in the sample surface, an impinging ion must be able to transfer enough kinetic energy to the surface atom to overcome its displacement threshold. Assuming a simple fully elastic knock-on event with a maximum energy transfer, a He ion needs a minimum kinetic energy of 130.2 eV to overcome the displacement threshold of ca. 20 eV^37^ of a Mo atom in MoS_2_. As the displacement threshold for S atoms is only ca. 6.9 eV,^38^ a minimum kinetic energy of 17.9 eV is already enough to produce a V_1S_ defect. Therefore, the He ions with a mean kinetic energy of 171 eV are able to introduce defects into both sublattices.

**Figure 1:**
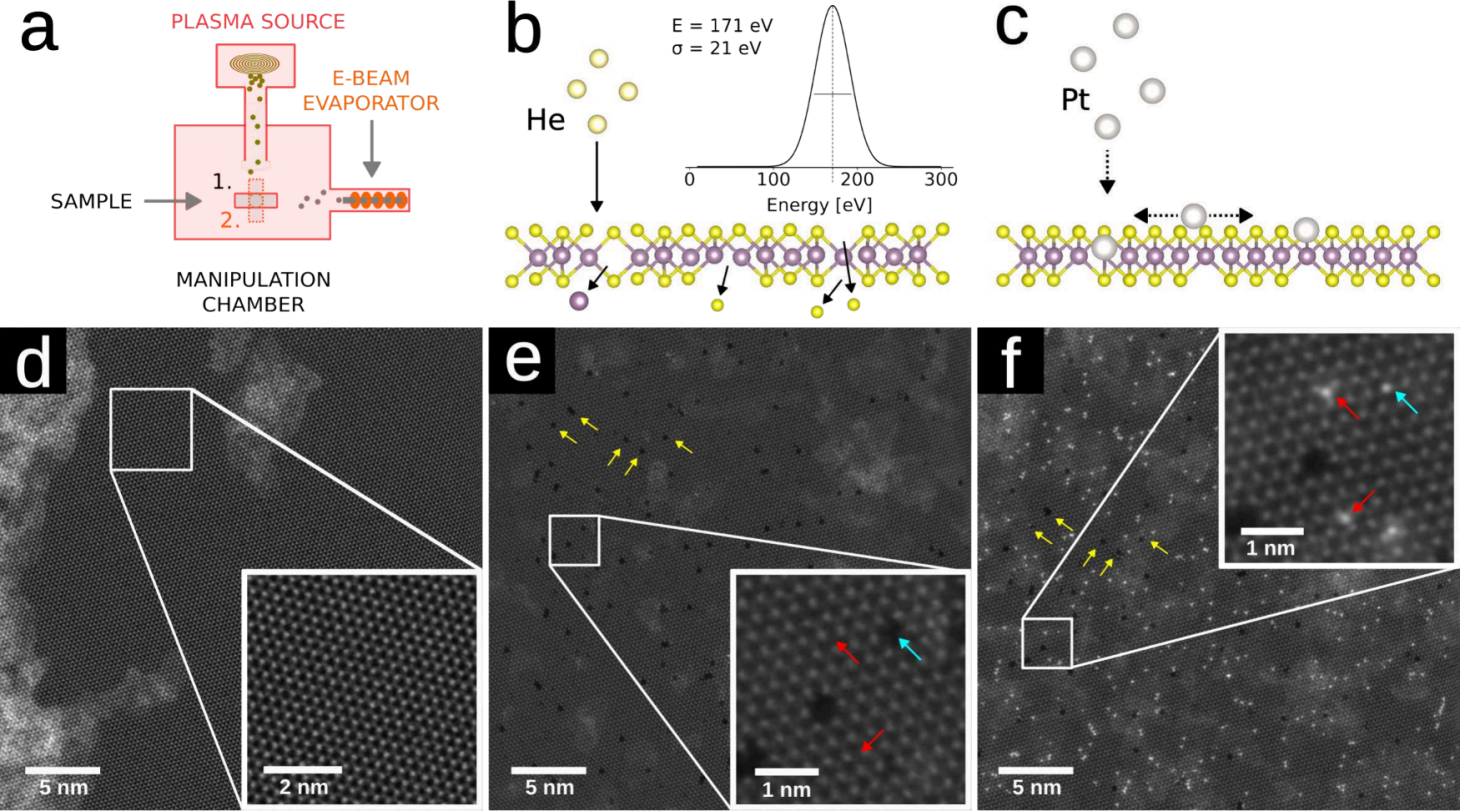
a) Schematic illustration of the sample manipulation chamber used for the study. In step 1 the sample is subjected to the ion beam, and in step 2 the sample is in the field of view of the e-beam evaporator. b) Schematic illustration of the defect-engineering process. The inset shows the beam energy profile of the He ions. (The full *dI*/*dV* curve can be found in Supporting Information Figure S1.) c) Schematic illustration of the single atom evaporation process. d) HAADF-STEM image of a clean MoS_2_ area before modification steps (not the same area as in the following images). e) HAADF-STEM image of MoS_2_ after 10 min irradiation with He ions. The red and turquoise arrows in the inset indicate V_1S_ (red) and V_Mo_ (turquoise) and defect sites that will be filled with Pt atoms; the yellow arrows mark the same defect features before and after Pt evaporation. f) HAADF-STEM image of roughly the same area and field-of-view as in panel e (see yellow arrows); the sites filled with Pt atoms are indicated with the red and turquoise arrows in the inset.

After 10 min of ion irradiation with an estimated total fluence of 1.25 × 10^13^ cm^–2^ the samples were transported under UHV to the microscope to image the defect structures. After imaging, Pt atoms were evaporated onto the sample using an e-beam evaporator while keeping the sample under UHV. Nearly exactly the same sample area is shown in Figure 1e before and in Figure 1f after the evaporation. It is evident that Pt atoms are incorporated into the MoS_2_ lattice, occupying the former V_1S_, V_2S_, and molybdenum vacancy (V_Mo_) sites.

HAADF and SSB images of defect structures are shown in Figure 2a together with corresponding image simulations conducted with *ab*TEM^39^ based on relaxed atomic models. A comparison of the HAADF intensities of V_1*S*_ and V_2*S*_ and the pristine S_2_ sites shows that it is difficult to differentiate between the pristine S_2_ sublattice and S vacancies in HAADF imaging, requiring high doses and high magnification, which come with the disadvantage of introducing additional defects during the imaging process. Fortunately 4D-STEM ptychography has a much higher contrast and dose efficiency compared to HAADF images.^33^ For 4D data collection, stacks with 512 × 512 real-space pixels were collected with a dwell time of 20 μs and an average dose of ca. 1 × 10^5^ e^–^/Å^2^. The phase information was retrieved using the single-sideband (SSB) method with post-acquisition aberration correction as described in ref 30.

**Figure 2:**
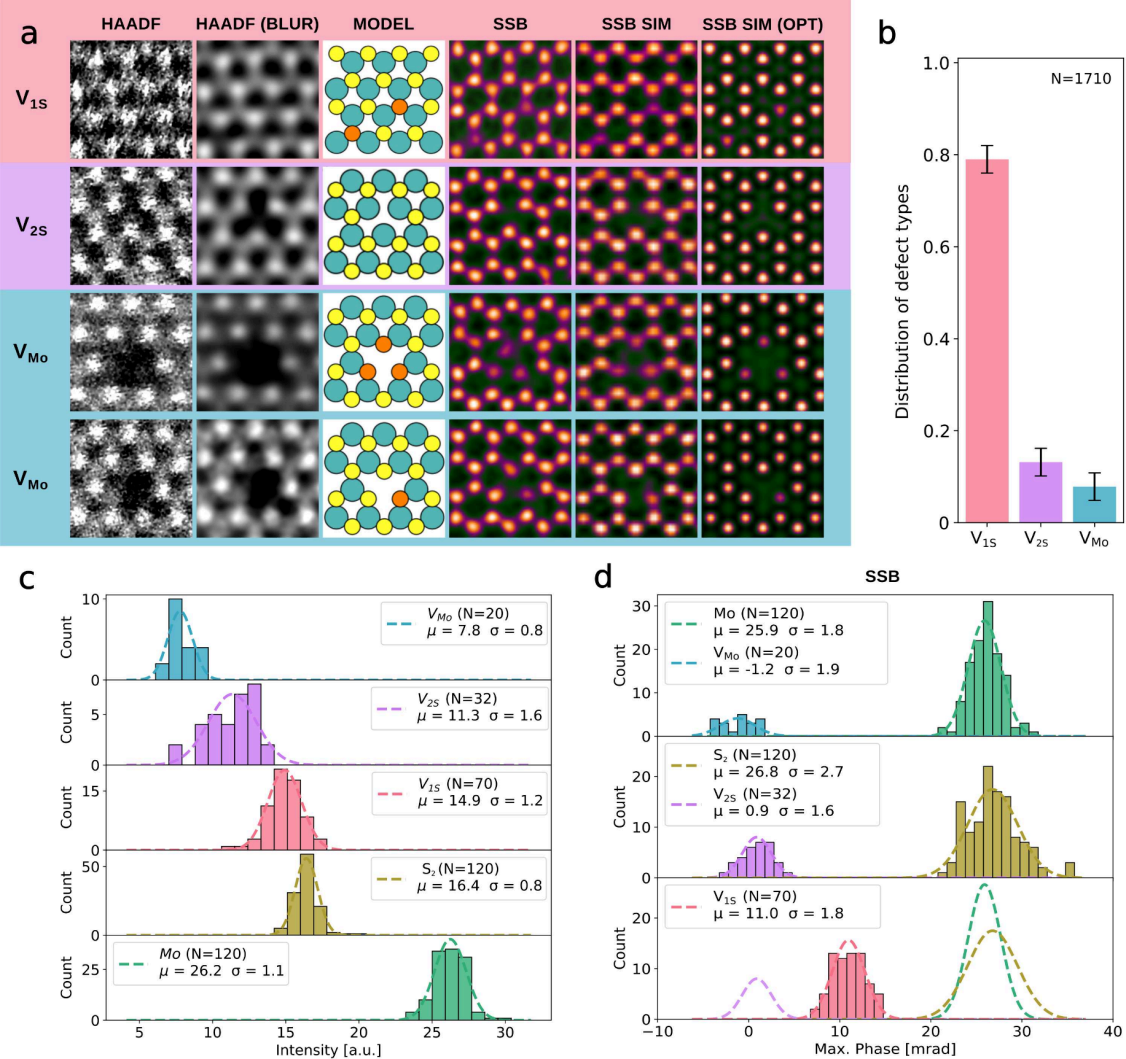
a) HAADF-STEM images (field of view ca. 1 nm) of defect structures without and with Gaussian blurring, atomic models of the imaged structures, SSB reconstructions of the phase information at the same location, as well as simulations of the SSB images corresponding to experimental parameters. The last column contains simulations of the SSB images under perfect conditions (unlimited dose, no residual aberrations). b) Relative occurrence of different defect types in the defect-engineered MoS_2_ based on SSB and HAADF images. The uncertainty in the columns is based on the variation between the observed images. c) Histograms of HAADF intensities at the Mo and S_2_ sublattice sites. The type of S vacancies (no vacancy, V_1S_, V_2S_) is determined using the SSB intensity at the respective S sublattice site. d) Histograms of SSB phase values at the same atomic sites as in c), together with Gaussian fits of the phase distribution of all structures. The μ in the legends indicates the center of the Gaussian fits, the σ is the standard deviation of the respective Gaussian, and *N* is the number of cases for each histogram.

The last two rows of Figure 2a contain examples of defect clusters around a V_Mo_ site. While in the HAADF images the neighborhood of the Mo vacancy is quite ambiguous and the number of neighboring S vacancies is hardly determinable, the SSB images clearly show a well-defined atomic structure. Mo–S vacancy structures with one to five missing sulfur atoms and a number of different vacancy configurations can be observed. Due to the relatively low ion fluence used in the experiments, the appearance of these vacancy structures is most likely due to a single impact and following collision cascades.

Figure 2b shows the ratio of the defect numbers derived from both large-scale HAADF imaging and small-scale phase reconstructions. Over 80% of the introduced defects are V_1S_, whereas V_2S_ and V_Mo_ contribute with ca. 12% and 8% to the overall defect density of 1.3 ± 0.4 defects per nm^2^, in agreement with what we would expect at this ion energy.^40^

Figure 2c,d contains histograms of the HAADF intensity and phase maxima at the same atomic positions in defect-engineered MoS_2_ as well as Gaussian fits of the distributions. The HAADF intensity distributions of pristine S_2_, V_2S_, and V_1S_ overlap significantly with each other and form a near-uniform distribution. By contrast, SSB images exhibit significantly larger phase ratios between the atomic columns, enabling more precise defect identification. The mean phase of the V_1S_ site is 11.0 ± 0.6 mrad, while the mean phase of the S_2_ site is 26.8 ± 0.7 mrad, which leads to an average phase ratio of 2.4 between the two cases, allowing for precise discrimination between defective and pristine sites in the sulfur sublattice. Additionally, the V_2S_ site with its mean phase of 0.9 ± 1.3 mrad can be easily discerned from both V_1S_ and S_2_. The uncertainties given for the mean phases correspond to the 95% confidence interval.

The distributions of V_2S_ and V_Mo_ are both centered near a phase of zero. Even though this seems to match optically with the simulated images, there is a significant difference visible in the line profiles of these structures (Supporting Information Figure S3). Unlike in HAADF imaging, the SSB phase displays a negative phase halo around a single atom, which converges to the background value of zero phase after some distance.^41^ In the case of MoS_2_, the negative halos of the six atomic columns around one hexagon nearly overlap, creating a deep phase trench with a small spike in the center of the hexagon. As only a few of the observed V_2S_ have a negative phase maximum, we suspect that the sites are actually filled with light elements, which has already been discussed by Yin Wen.^42^ Supporting Information Figure S3c,d contains image simulations and corresponding line profiles of V_2S_ doped with C and O, which are in a good agreement with the observed phase maxima at the supposed V_2S_ sites. Additional evidence stems from the fact that an unfilled V_2S_ would be subject to a lattice contraction of up to 12%,^43^ which is not observed here. These substitutions are most likely C atoms from the hydrocarbon contamination which diffuse freely on the MoS_2_ surface due to their low diffusion barriers (0.56 eV for C,^44^ in comparison 1.92 eV for O^45^), before they fall into an energetically more favorable V_2S_ vacancy site (binding energy of 4.5 eV^44^).

Even though similar reasoning could be applied to the observed phase maxima at V_Mo_ sites, the line profile analysis in Supporting Information Figure S4a,b shows that the center position of the V_Mo_ sites has a local maximum that is much lower than expected for V_Mo_ filled with a C atom in Figure S4d. Therefore, we think that the maximum values plotted in Figure 2c indeed correspond to unfilled V_Mo_. A notable exception is shown in Supporting Information Figure S4c, where the V_Mo_ is clearly filled with a heteroatom, which we assume to be a S atom based on the very similar contrast to the neighboring S atoms.

Due to the very similar phases of pristine Mo with S_2_ sites, as well as V_Mo_ with V_S2_ sites, it is difficult to determine the sublattices from SSB images alone. This is, however, not an issue, as the concurrent signal from HAADF (or virtual ADF) imaging provides sufficient contrast to distinguish between the Mo and S_2_ sublattices. While in HAADF images the presence of thin hydrocarbon contamination on the MoS_2_ surface and at vacancy sites is indicated only by a low increase in background intensity, SSB reconstruction allows the visualization of individual carbon atoms in the vacancy sites and, to some degree, also on the MoS_2_ surface (for an example see Supporting Information Figure S5). The phase variations introduced due to these light element adatoms, together with the overlapping negative halo effect on nearby atoms,^41^ residual aberrations, scan distortions, and shot noise due to the limited dose,^33^ are probably the main contributors to the observed phase variations of up to 3 mrad in all phase measurements.

As was already visible in Figure 1f, after Pt evaporation, most Pt atoms occupy the sites of the sulfur sublattice. Some of these Pt atoms are unstable under the electron beam and jump to another sulfur site once the scan reaches their position (see Supporting Information Figure S6). These are most probably not adatoms on the pristine surface, because the energetically more favorable site for a Pt adatom is on top of a Mo site (Figure 3a). Further, the surface diffusion migration barriers for Pt are only between 0.4 and 0.6 eV (see Figure 3a,b) depending on the location of the adatom, and thus, the Pt atoms can easily diffuse over the MoS_2_ surface until they fall into an energetically more favorable vacancy site. Therefore, we assume that the observed atom jumps take place between S vacancy sites, as described in ref 23. The diffusion energy paths of Pt atoms into V_1S_, V_2S_, and V_Mo_ vacancies are depicted in Figure 3b–d and show binding energies of 2.6, 2.5, and 4.6 eV, respectively. All these binding energies are sufficient to ensure the stability of the implanted atom at room temperature.

**Figure 3:**
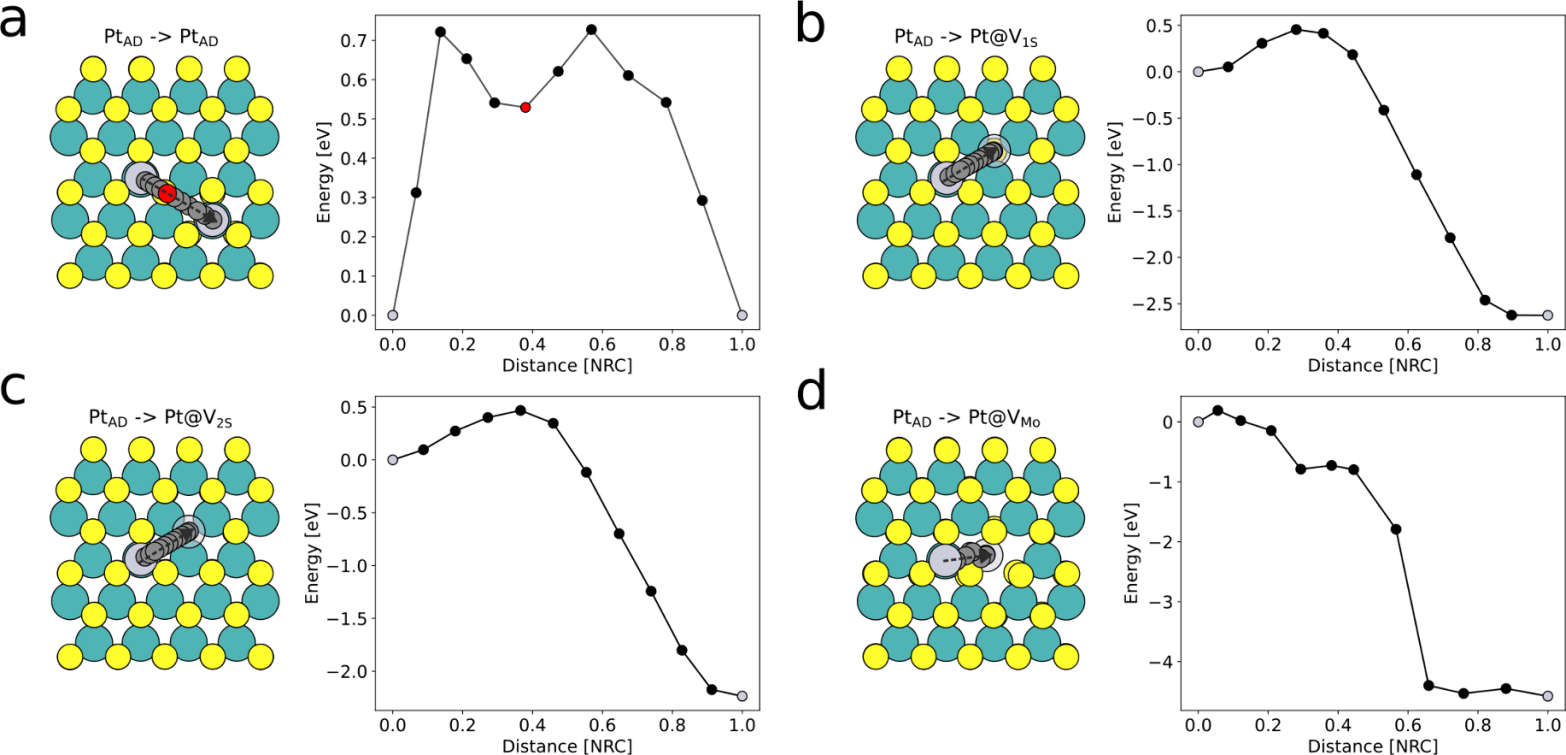
a–d) Diffusion paths of Pt atoms on the surface calculated by the nudged elastic band method. The *x*-axis in the energy diagrams is given in relative atomic mass-weighted distances (normalized reaction coordinates, NRC). The black dots in the energy diagram match the (semitransparent) gray circles on the atomic model. Start and end positions of the diffusion process are marked with silver dots. a) Diffusion from the top of a Mo site to the top of another Mo site over the metastable position on top of a S site (marked by the red dot). b) Diffusion from the surface to a V_1S_ site, c) to a V_2S_ site, and d) to a V_Mo_ site.

Experimental and simulated HAADF and SSB images of the most typical Pt-doped sites are shown in Figure 4a. As Pt atoms have a significantly higher nuclear charge (*Z* = 78) than the surrounding Mo (*Z* = 42) and S (*Z* = 16) atoms, the Pt atom in the HAADF images appears as a large bright feature, obscuring the neighboring atomic structure. The contrast difference between a Pt atom located at a V_2S_ site (Pt@V_2S_) with a theoretical Pt/Mo intensity ratio of 2.5 is very similar to that of Pt@V_1S_ with a theoretical (S+Pt)/Mo intensity ratio of 2.7. The SSB images give a much clearer picture, with a Pt/Mo phase ratio obtained by simulations of 1.19 and a respective simulated (S+Pt)/Mo phase ratio of roughly 1.85. This is reflected in the experimental data, where we observed an average phase of 43.4 ± 1.6 mrad for Pt@V_1S_, 33.7 ± 1.1 mrad for Pt@V_2S_, and 33.8 ± 1.9 mrad for Pt@V_Mo_, which results in Pt/Mo and (S+Pt)/Mo ratios of 1.2 and 1.6, respectively. SSB imaging is also a powerful tool for analyzing Mo substitutions. Due to the considerably lower phase ratio of the Pt atom in comparison to the neighboring sites, discerning the neighborhood and exact placement of the Pt atom implanted in the defect clusters around V_Mo_ sites becomes significantly more straightforward, as can be seen in the third row of Figure 4a.

**Figure 4:**
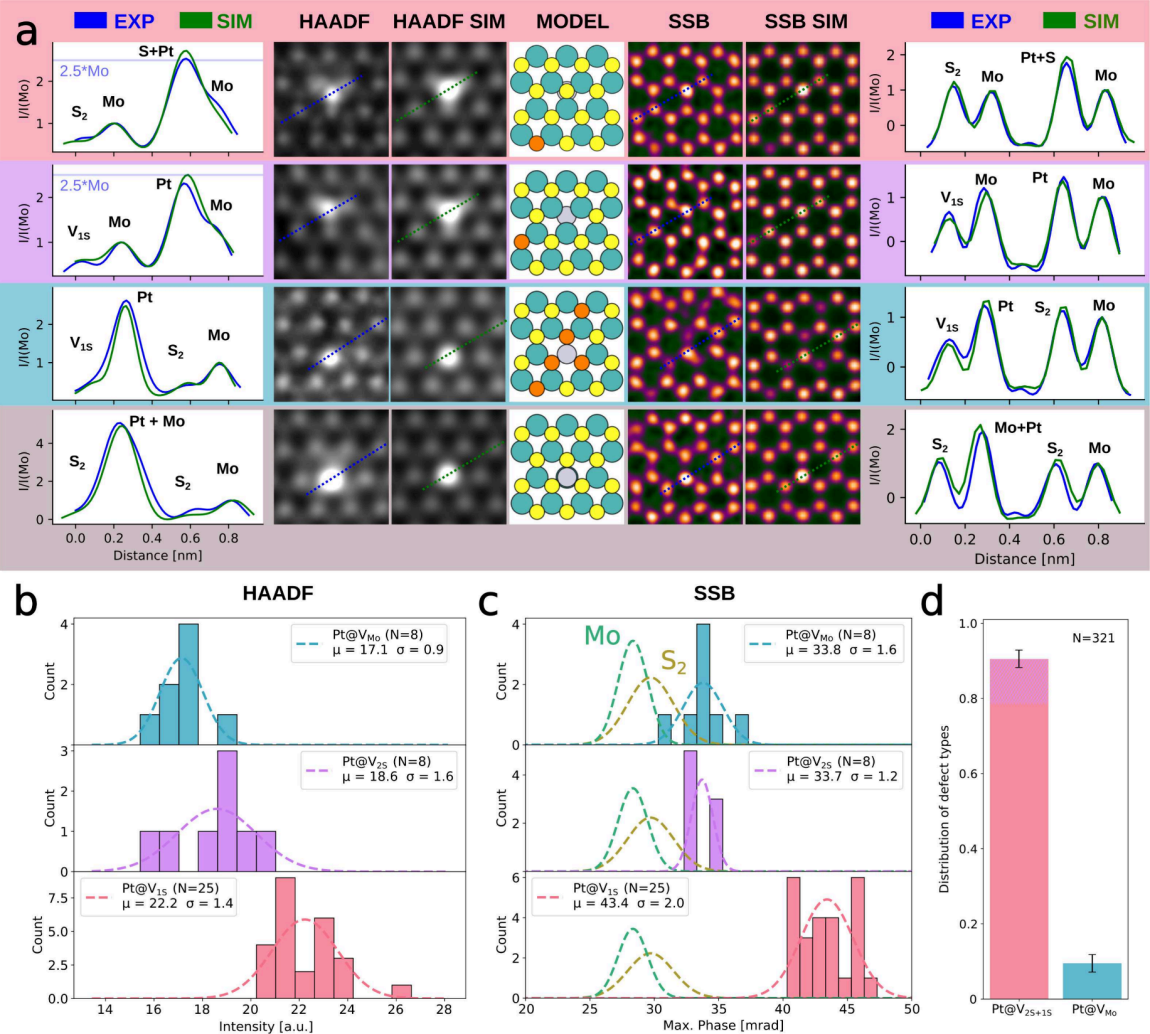
a) Line profiles of Gaussian blurred HAADF-STEM images of dopant structures, shown alongside the blurred images as well as HAADF image simulations, atomic models of the imaged structures, SSB reconstructions of the phase information at the same location, and realistic simulations of the SSB images. From top to bottom, the rows contain data of Pt@V_1S_, Pt@V_2S_, Pt@V_Mo_, and Pt adatoms. b) Histograms of HAADF intensities at Pt@V_Mo_, Pt@V_2S_, and Pt@V_1S_ sites and Gaussian fits of the distributions. The types of Pt substitutions at the S_2_ sublattice are determined using the SSB phase at the respective locations. c) Histograms of SSB phase values at the same atomic sites as in b), together with Gaussian fits of the phase distribution of the structures. The violet and turquoise lines represent the distributions of the Mo and S_2_ phase values, respectively. The μ in the legends indicates the center of the Gaussian fits; the σ is the respective standard deviation. d) Relative occurrence of dopant structures obtained from large-scale HAADF imaging. As Pt@V_1S_ and Pt@V_2S_ are barely distinguishable in HAADF images, both types are counted together and the tip of the Pt@V_1S+2S_ column is shaded in purple to mark the approximate ratio of Pt@V_2S_ based on the ratio between V_1S_ and V_2S_. The uncertainty in the columns is based on the variation between observed images and is rounded up to the next integer percentage. *N* is the number of cases for each histogram.

Unfortunately the complex contrast formation in SSB may lead to misinterpretation of Pt adatoms located on top of the Mo sublattice, as can be seen in the fourth row of Figure 4a, where HAADF imaging of this configuration provides significantly better contrast with a theoretical (Pt+Mo)/Mo intensity ratio of 5.6 compared with a Pt/Mo ratio of 2.5. As the observed adatoms are not displaced by the electron beam, it is safe to assume that these Pt atoms are stabilized by a very thin carbon contamination on the MoS_2_ surface.

Figures 4b and 4c show histograms of the maximum HAADF intensities and phase maxima at the same Pt-doped sites. While Pt@V_1S_ and Pt@V_2S_ distributions overlap in HAADF imaging, the phase distributions in SSB imaging are significantly more distinct. As we have obtained only a limited amount of SSB data, the distribution of different Pt dopant types plotted in Figure 4d is based on large-scale HAADF images. Due to the poor distinguishability of Pt@V_1S_ and Pt@V_2S_ in HAADF, these are shown in the same column. Notably, the ratio between Pt atoms in S and Mo vacancies is the same as the ratio of the vacancies themselves, leading to the assumption that the Pt atoms are incorporated into the first defect they find after landing on the MoS_2_ surface. Therefore, we shaded the tip of the Pt@V_1S+2S_ column in Figure 4d in purple to mark the approximate ratio of Pt@V_2S_ based on the ratio between V_1S_ and V_2S_.

Since the phase contrast is directly related to the local electron charge density, which can change depending on the chemical interactions between the atoms, using a model of noninteracting atoms is, strictly speaking, not sufficient for fully quantitative image simulations.^46,47^ Supporting Information Figure S7 contains simulated images with independent-atom-model (IAM) and DFT potentials of Pt@V_1S_ and Pt@V_2S_ structures as well as their difference. Evidently the simulated phase shifts due to charge transfer to the Pt dopant sites in MoS_2_ are in the range of one percent of the absolute phase values and thus well within the range of the observed phase uncertainty in SSB. This suggests that simulations based on the IAM are precise enough for qualitative analysis in this system.

In summary, by combining helium ion irradiation to create controlled vacancy defects and subsequent Pt atom incorporation via evaporation, we successfully achieved substitutional platinum doping of sulfur (V_1S_, V_2S_) and molybdenum (V_Mo_) lattice sites in monolayer MoS_2_. We further demonstrated that SSB is a powerful imaging technique for reliably identifying and characterizing defect and dopant structures in Pt-doped MoS_2_ monolayers at an atomic resolution. The phase contrast obtained by SSB allows reliably differentiating between various dopant and defect configurations, such as Pt atoms in single and double sulfur vacancies, which are difficult to resolve using HAADF-STEM imaging alone.

However, SSB is not without limitations: its phase contrast depends on the local atomic environment, there is a reduced *Z*-contrast between heavy and light elements, and further nonlinearity can be introduced due to charge redistribution, all of which can make image interpretation challenging. It is therefore vital that SSB studies of modified 2D materials should be assisted by quantitative image simulations. It should also be noted that in some systems SSB imaging does not provide any advantage over traditional imaging techniques, e.g., MoS_2_ co-doped with two similar high *Z*-elements such as Au and Pt. Further, the larger data volumes and computational demands required for SSB make it less scalable for large-area imaging compared to HAADF-STEM.

In the future, our substitutional doping method could be further optimized by fine-tuning the ion beam parameters to increase the precision of the defect creation process. Moreover, a resulfurization step could be applied before or after Pt evaporation to repair undesired defects, thereby improving the control and uniformity of dopant placement. These refinements enhance the scalability and reproducibility of Pt doping in MoS_2_, offering a promising pathway for controlled defect engineering and functionalization of materials for advanced applications in catalysis and electronics. Future experiments with substitutional dopants in MoS_2_ could benefit from advanced 3D structural characterization techniques, such as few-tilt ptychotomography,^48^ to further expand the potential of this approach for targeted material design.

## Supplementary Material



## Data Availability

The experimental data supporting these findings is available through the University of Vienna PHAIDRA repository https://phaidra.univie.ac.at/o:2093675. The SSB reconstruction code can be downloaded from https://gitlab.com/pyptychostem/pyptychostem.

## Notes

Additional information about the sample preparation and the substitutional doping method; details of the experimental imaging conditions; remarks on contamination and EEL spectra; details of the image simulations as well as information about DFT calculations; supporting figures of the implantation process as well as an example diffraction pattern. (PDF)
