# Supplementary material for: Uncovering the Atomic Structure of Substitutional Platinum Dopants in MoS2 with Single-Sideband Ptychography
Source: Nano Lett. 2025 May 23;25(22):8931–8. doi: 10.1021/acs.nanolett.5c00919 (PMC12142672; doi:10.1021/acs.nanolett.5c00919)
Supplement: Supplementary file 1 [file nl5c00919_si_001.pdf]

Supporting Information: Uncovering the atomic structure of substitutional platinum dopants in MoS_2 with single-sideband ptychography

David Lamprecht,[*,†,‡] Anna Benzer,[†] Manuel Längle,[‡] Mate Capin,[†] Clemens Mangler,[‡] Toma Susi,[*,‡] Lado Filipovic,[†] and Jani Kotakoski[*,‡]

†*Institute for Microelectronics, TU Wien, Gußhausstraße 25-29, 1040 Vienna, Austria*

‡*University of Vienna, Faculty of Physics, Boltzmanngasse 5, 1090 Vienna, Austria*

E-mail: lamprecht@iue.tuwien.ac.at; toma.susi@univie.ac.at; jani.kotakoski@univie.ac.at

Methods

Sample preparation The MoS_2 sample was grown on SiO_2 via chemical vapor deposition (CVD)[1] using liquid-phase exfoliated MoO_3 as precursor and consists of mostly triangular monolayered flakes with an edge lengths between 5 and 20 μm. The samples were subsequently transferred in air onto a gold transmission electron microscopy grid with a holey membrane of amorphous carbon (Quantifoil R 1.2/1.3 Au grid) using the method described in Ref. 2.

Substitutional doping with single Pt atoms After transferring to the TEM grid, the MoS_2 samples were introduced into the interconnected near-UHV CANVAS system[3] (base pressure of 10^{-8} mbar), which features both a SPECS ECR-HO microwave plasma

generator and evaporation sources. Low-energy He ions from the plasma generator with a current of ca. 2.5 nA at a He partial pressure of ca. 2.5×10^{-5} mbar were used to irradiate the MoS_2 samples. The measured ion energy for these parameters is approximately normally distributed with a mean of ca. 171 eV and a standard deviation of ca. 21 eV. Irradiation for 10 min corresponds to a fluence of ca. 1.25×10^{13} cm^{-2}. The plasma treatment was followed by evaporation of the platinum. For Pt, the tip of a 99.9% Pt rod was heated up with an EFM-3 e-beam evaporator using a filament current of 2.9 A and an extraction voltage of 1850 V to produce a Pt flux of ca. 0.6 nA. After 10 min of evaporation, single Pt atoms were found in the MoS_2 lattice.

Microscopy and spectroscopy After implantation, the samples were transferred inside the UHV system to the aberration-corrected Nion UltraSTEM 100 scanning transmission electron microscope operated at 60 kV acceleration voltage with a beam current of ca. 40 pA. Images were acquired using a HAADF detector with a probe convergence semi-angle of ca. 35 mrad and a semi-angular range of 80–300 mrad. HAADF images with 2048×2048 pixels were further processed using Gaussian blurring in order to reduce noise and increase contrast of the images. EEL spectra were recorded with a Gatan PEELS 666 spectrometer with an Andor iXon 897 CCD camera and an energy-dispersion of 0.5 eV/pixel.[4] For simultaneous 4D data collection, stacks with 512×512 real-space pixels were collected using a Dectris ARINA direct-electron detector (192×192 pixel) with a dwell time of 20 μs and an average dose of ca. 1×10^5 e$^-$/Å^2. The direct-electron detector falls completely within the HAADF detector and the maximum scattering angle collected by the detector is 36 mrad. To reduce the size of the data, recorded at each imaged position (which can be up to 10 GB for a single dataset), we reduced the size of the convergent-beam electron diffraction patterns recorded at each probe position to 48×48 pixels by 4 times binning, which only has a negligent influence on the quality of the reconstructed phase.[5] After binning the reciprocal pixel size is approximately 1.5 mrad/pixel. SSB was performed with the open-source PyPtychoSTEM package,[6] using the experimental 4D-STEM data as input. The convergence angle was set to 35 mrad and

the step size was 0.156 Å per pixel (matched in the SSB reconstruction). Post-collection aberration correction was applied using singular value decomposition to identify the residual aberrations which were then counteracted.

Density functional theory To determine the minimum energy paths and transition states for several possible diffusion events on the MoS_2 monolayer, a density functional theory (DFT) based *ab initio* simulation approach as implemented in the CP2K[7] code (version 2023.2) was used. For all calculations the norm-conserving, separable, dual-space Gaussian-type pseudopotentials of Goedecker, Teter, and Hutter (GTH)[8] were used with a 500 Ry plane wave cutoff of the multigrid. The exchange-correlation interactions where treated with the Perdew–Burke–Ernzerhof (PBE)[9] generalized gradient approximation. As a basis for all calculations, $5 \times 5 \times 1$ MoS_2 monolayer supercells were used consisting of 75 atoms with a vacuum layer of 40 Å in order to prevent artificial interactions between periodic images. After a cell relaxation of the pristine material, the supercells were modified in order to determine the minimum energy paths for diffusion of Pt and S atoms on the surface of (defective) MoS_2. For this, the climbing image nudged elastic band method[10] was used with 12 images and a maximum force convergence criteria of 0.01 Hartree per Bohr.

HAADF and 4D-STEM image simulations HAADF-STEM image simulations were performed based on the DFT-relaxed models using the *ab*TEM package.[11] Similar to the experiment, the HAADF detector semi-angular ranges was set to 80–300 mrad and the probe convergence angle was set to 35 mrad. For all simulations an electron beam energy of 60 keV was assumed. To account for thermal diffuse scattering, we implemented the frozen-phonon model with 20 snapshots per image using standard deviation of atomic displacements values taken from Ref. 12. For 4D-STEM the same parameters were used, but instead of the HAADF detector a pixelated detector setting was used. After creating the artificial 4D-STEM data, the phase images were reconstructed using the same algorithm and parameters as in the corresponding experimental images, including the dose per area simulated by adding Poisson noise to the diffraction patterns. To account for finite probe-size effects in SSB

images, we added a Gaussian blur over the simulated images to match the line profile of the experimental SSB data. For the charge-transfer simulations, the DFT potential was calculated from the all-electron charge density converged with GPAW, as described in Ref. 13.

Evaporation of Pt on graphene and EELS measurements Commercial graphene grown via CVD provided on a sacrificial polymethyl methacrylate (PMMA) layer (Easy Transfer graphene) was transferred onto a SiN TEM grid with with 3 μm holes (Silson Ltd) via a liquid transfer method using deionized water as the carrier liquid. Following the transfer onto the substrate the graphene was heated on a hot plate at 150°C for 1 h. The PMMA sacrificial layer was then removed via an acetone bath at 50°C for 1 h, after which the grid rested in isopropyl alcohol (IPA) at room temperature for another hour. Graphene samples were cleaned using a 6 W continuous wave diode laser with a wavelength of 445 nm with a spot size of 0.3×1.5 mm^2 as described in Ref. 14. The surface was illuminated at 27% of the maximum power for 6 min multiple times, where the power was set by changing the duty cycle. With these parameters, not all of the surface contamination is removed, but large-enough atomically clean patches are created for acquiring statistically meaningful data. Pt was then evaporated onto the sample for 15 minutes with a flux of 0.25 nA.

Remarks on contamination

A significant number of the evaporated metal atoms can also be found on or next to hydrocarbon contamination (brighter, diffuse contrast on the image). Most of these atoms are still isolated and perhaps form chemical bonds with the hydrocarbons,[15] but are not incorporated into the lattice.

As is evident from the images in main article Fig. 1 d-f, ion irradiation and metal evaporation lead to a slight increase in undesired hydrocarbon contamination of the sample, effectively covering vacancy sites and incorporated metals. The same can be expected for

exposure of the defective MoS_2 surface to ambient conditions during sample transfer. To allow efficient implantation of dopants, protecting the sample from ambient between the steps is therefore crucial for the procedure. Nevertheless, even in UHV, every modification step can create additional contamination. Contamination during evaporation can be minimized by heat-treating the Pt source and cleaning the modification chamber with O_2 plasma (see Supplementary Material, Fig. S2).

EELS spectra of the evaporated material

Eeven though the high Z-contrast of the dopant atoms in HAADF-STEM and the excellent agreement with SSB image simulations are strong evidence for the element of the implanted atoms, definitive proof of the implantation of Pt atoms could benefit from additional spectroscopic evidence. Unfortunately, we were not able to obtain single-atom electron-energy loss (EEL) spectra of the Pt O_2 edge (the only Pt edge accessible to our EEL spectrometer) due to the limited stability of the MoS_2 under the electron beam and the low cross section of the Pt edge. Nevertheless, to provide spectroscopic evidence of the evaporated material, we evaporated Pt atoms onto graphene using the same setup and similar parameters. Supplementary Material Fig. S8 shows EEL spectra of a small Pt cluster formed in the graphene lattice, unambiguously demonstrating the presence of Pt.

Supplementary Figures

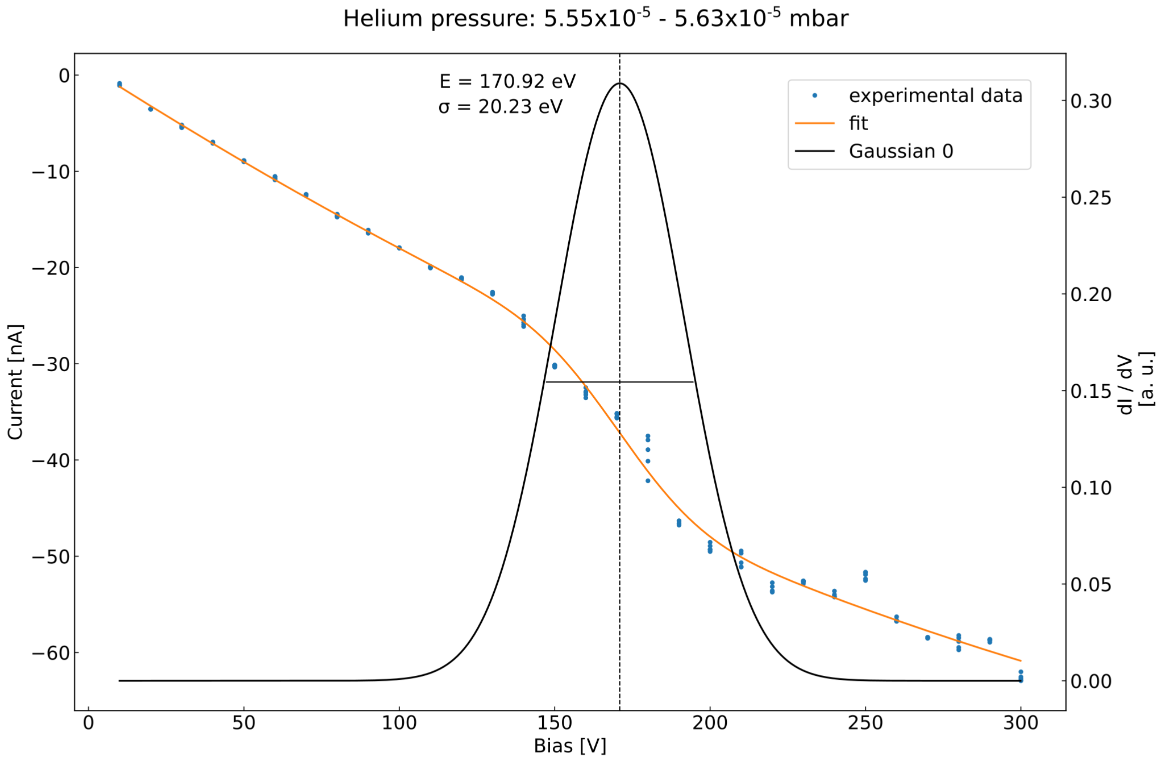

Figure S1: Beam profile and $\mathrm{d}I/\mathrm{d}V$ curve of the deceleration measurement of the ion plasma. The experimental details of this analysis can be found in.[16]

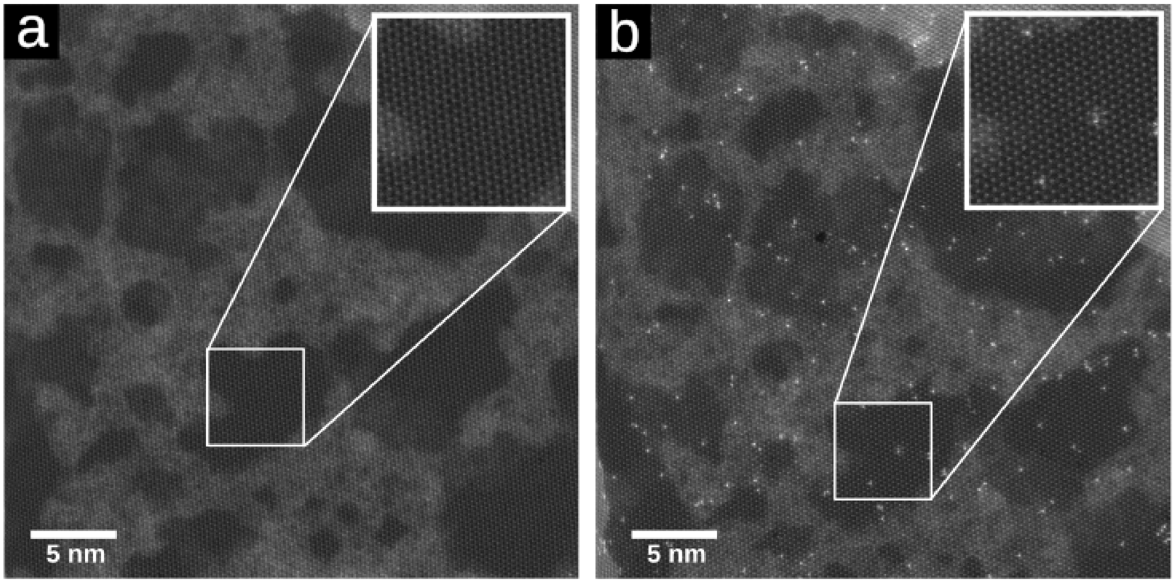

Figure S2: a) HAADF-STEM image of the pristine MoS_2 surface before evaporation b) HAADF-STEM image of the same area after evaporation of single Pt atoms.

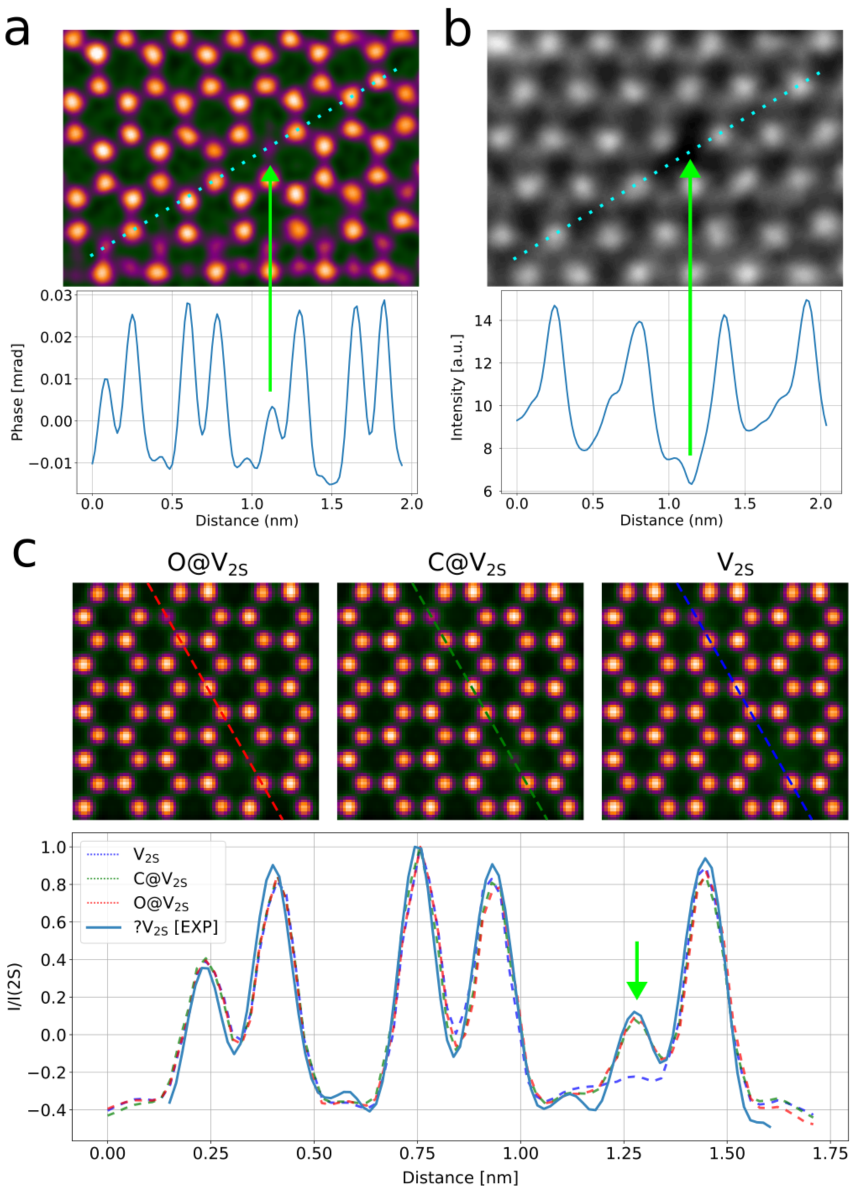

Figure S3: a) SSB phase image of highly defective MoS_2 next to a single vacancy defect line similar to the one reported in Ref. 17. The line profile over a V_{1S} and a V_{2S} is horizontally matched with the image. The unexpected local maximum at the V_{2S} is marked with a green arrow. b) Gaussian blurred HAADF-STEM image of the same structure with horizontally matched line profile. The HAADF-STEM contrast has a minimum exactly at the location of the V_{2S} (green arrow). c) Simulated SSB phase images of V_{2S} without heteroatoms, with a C dopant, and a O dopant. The plot below contains line profiles of the simulated structures and the experimental data from panel a. The location of the questionable V_{2S} site is marked with a green arrow in the line profile.

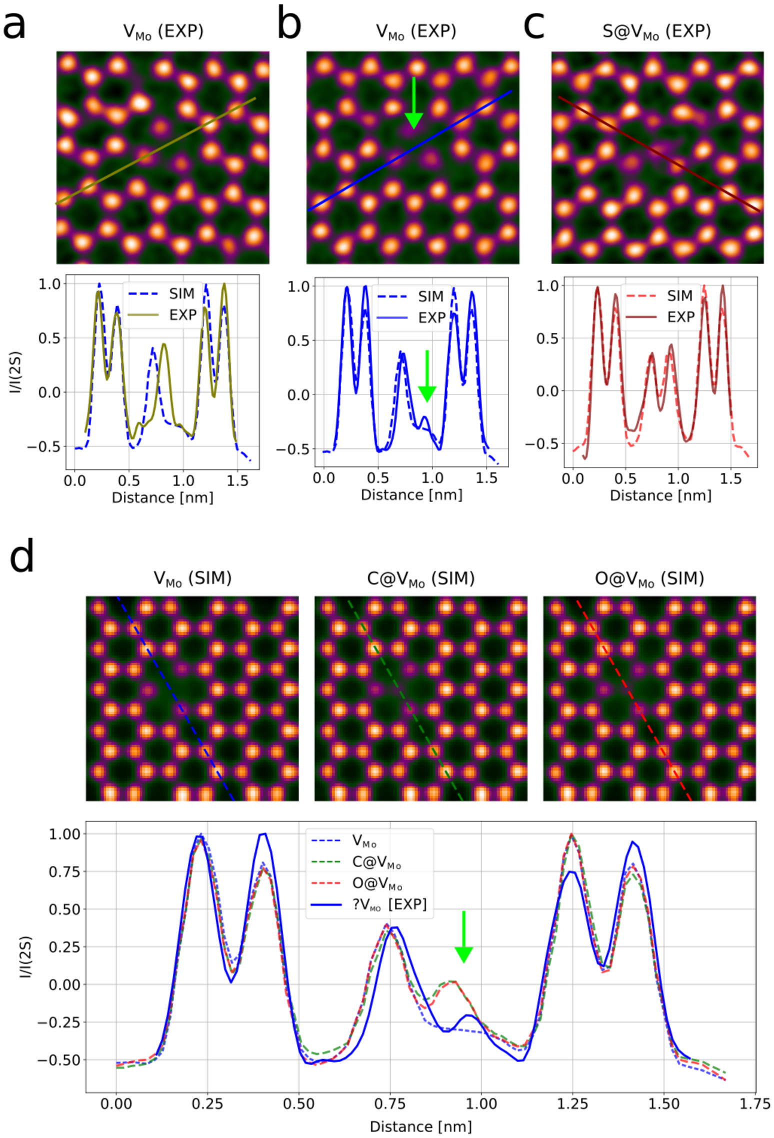

Figure S4: a) SSB phase image of a defect cluster with three S atoms missing around a V_{Mo} site. The simulated line profile corresponds to the first image in panel d of this figure. b) SSB phase of a similar defect cluster as in a. The simulated line profile corresponds to the first image in panel d. The unexpected local maximum at the V_{Mo} is marked with a green arrow. c) SSB image of a defect cluster with a S atom replacing the Mo atom. The simulated line profile corresponds to an S atom at the Mo position. d) Simulated SSB phase images of V_{Mo} surrounded by three V_{1S} without heteroatoms, with a C dopant and O dopant. The plot below contains line profiles of the simulated structures and the experimental data from panel b. The location of the questionable V_{Mo} site is marked with a green arrow in the line profile.

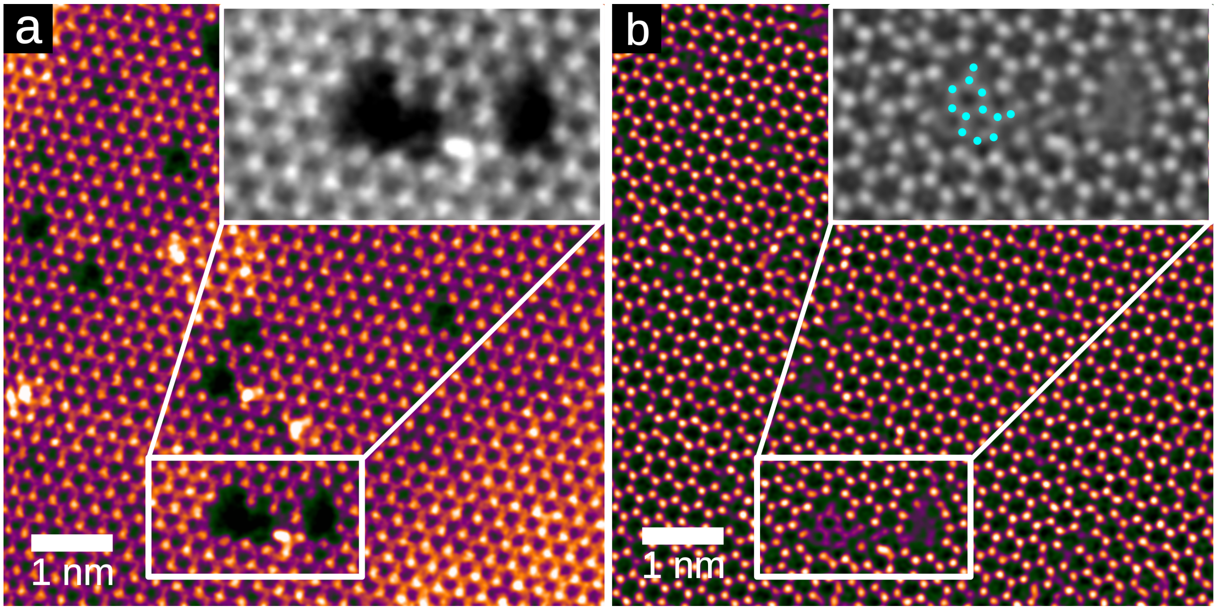

Figure S5: a) HAADF-STEM image of a defect cluster on contaminated MoS_2. b) SSB phase image of the same area: inside the defect cluster a hexagonal ring of *sp* hybridized carbon is clearly visible. The approximate positions of the carbon atoms in the defect cluster are marked in the inset with cyan dots.

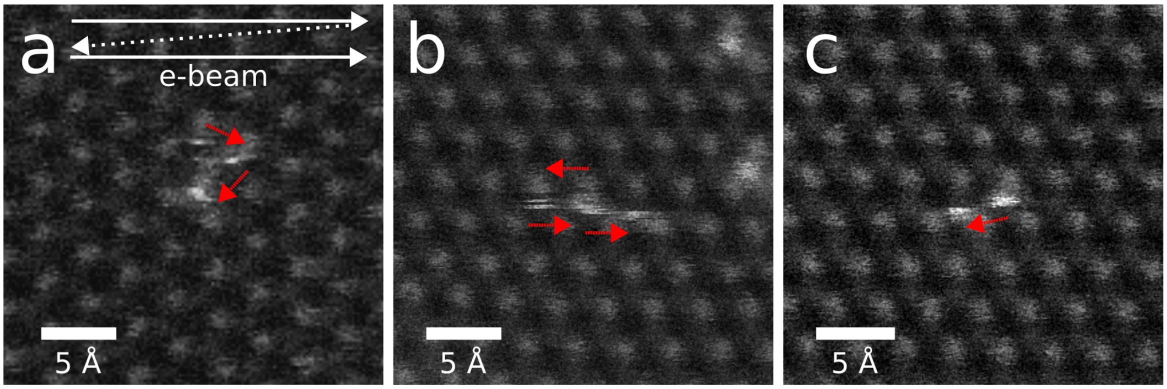

Figure S6: a-c) HAADF-STEM images of Pt atoms that are displaced from their position by the electron beam and imaged at different locations within the same frame. The trajectory of the Pt atoms is marked in red. The direction of the scanning electron beam (white lines) is overlaid in panel a).

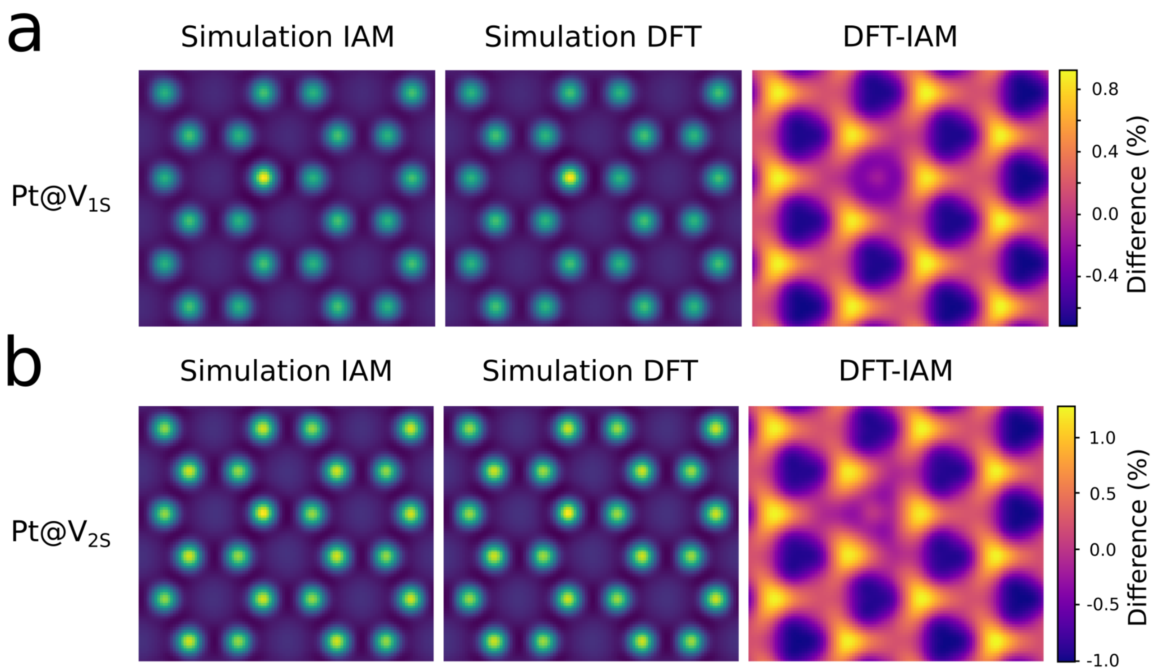

Figure S7: Phase image simulations of structures with potentials based on IAM and DFT as well as their difference for a) Pt@V_{1S} and b) Pt@V_{2S}.

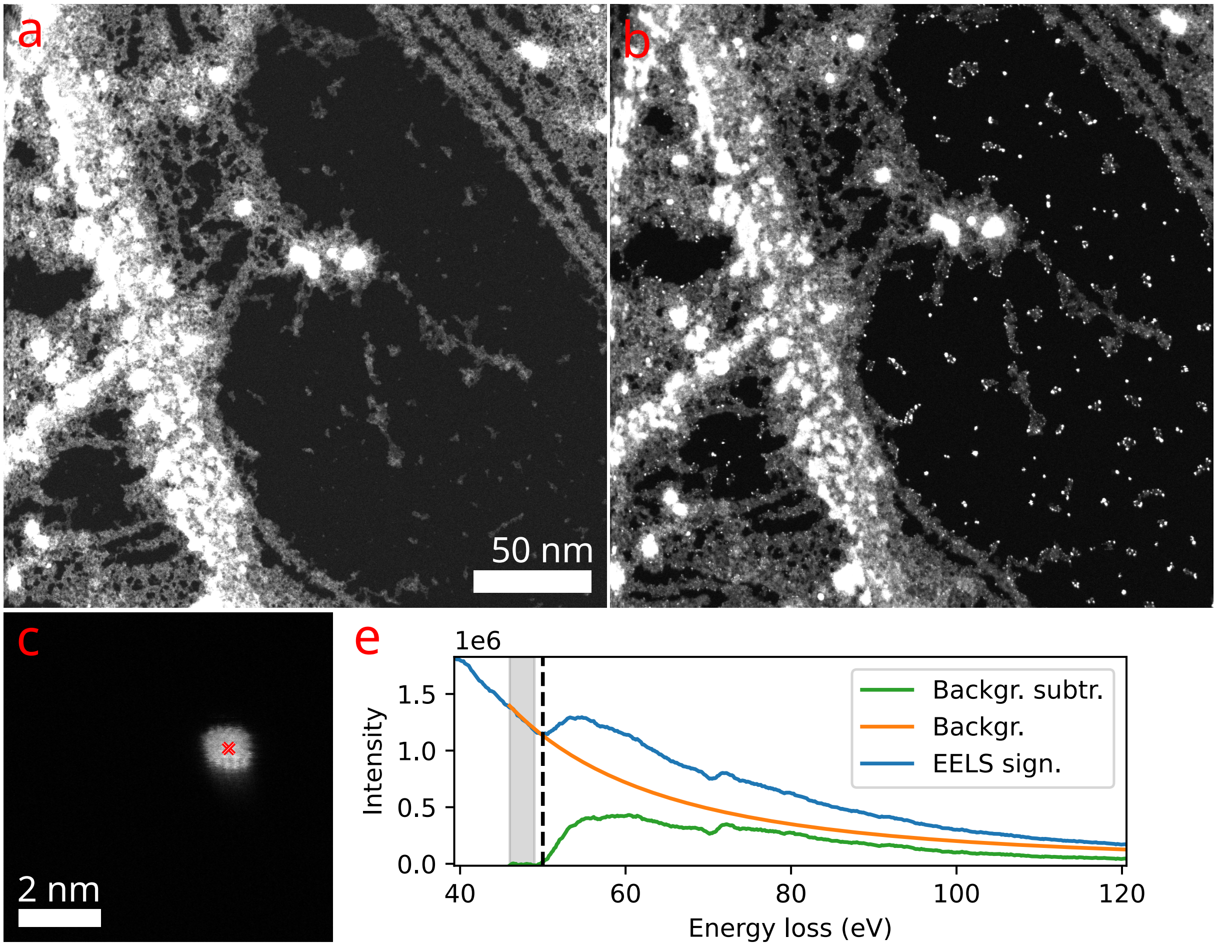

Figure S8: a) Overview HAADF-STEM image of laser-cleaned graphene. b) Overview HAADF-STEM image of the same area after Pt evaporation with an e-beam evaporator (flux: 0.25 nA, time: 15 min). c) Smaller field of view HAADF-STEM image of Pt cluster on graphene with the position of the EEL spectrum (shown in d) marked with a red cross. d) EEL spectrum recorded at the position of the red cross in panel c. The signal of the Pt cluster is shown in blue, with the background in orange and the background-subtracted spectrum in green. A power law is fitted to the original spectrum in the range of 46.0 to 49.0 eV (gray area). A black marker is positioned at an energy-loss of 50 eV. This corresponds to the Pt $O_{2,3}$ and Pt $N_{4,6}$ edges. Spectral analysis was done using hyperSpy.[18] The peaks align with reference spectra (EELS Atlas, https://eels.info/atlas/platinum). This also agrees closely with the spectrum shown in the Supplementary Figure S23 of Ref. 19

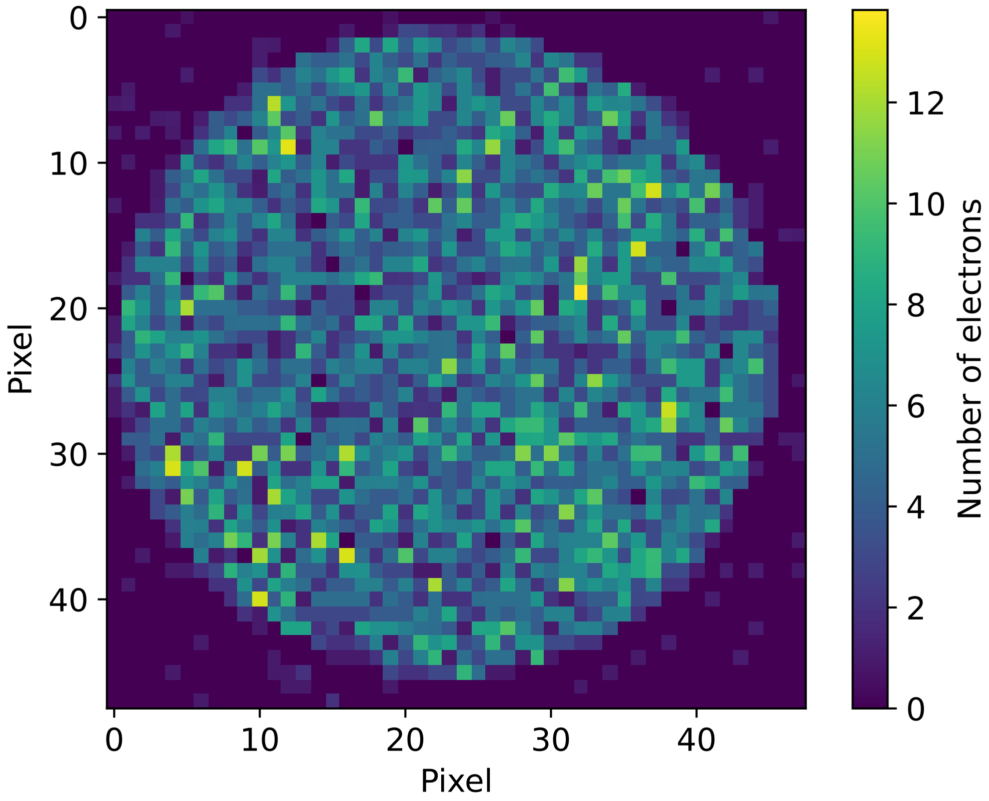

Figure S9: Typical convergent-beam diffraction pattern obtained with the Dectris ARINA direct-electron detector after 4-times binning.

References

(1) O'Brien, M.; McEvoy, N.; Hallam, T.; Kim, H.-Y.; Berner, N. C.; Hanlon, D.; Lee, K.; Coleman, J. N.; Duesberg, G. S. Transition Metal Dichalcogenide Growth via Close Proximity Precursor Supply. *Scientific Reports* **2014**, *4*, 7374.

(2) Meyer, J. C.; Girit, C. O.; Crommie, M. F.; Zettl, A. Hydrocarbon lithography on graphene membranes. *Applied Physics Letters* **2008**, *92*, 123110.

(3) Mangler, C.; Meyer, J.; Mittelberger, A.; Mustonen, K.; Susi, T.; Kotakoski, J. A Materials Scientist's CANVAS: A System for Controlled Alteration of Nanomaterials in Vacuum Down to the Atomic Scale. *Microscopy and Microanalysis* **2022**, *28*, 2940–2942.

(4) Susi, T.; Hardcastle, T. P.; Hofsäss, H.; Mittelberger, A.; Pennycook, T. J.; Mangler, C.; Drummond-Brydson, R.; Scott, A. J.; Meyer, J. C.; Kotakoski, J. Single-atom spectroscopy of phosphorus dopants implanted into graphene. *2D Materials* **2017**, *4*, 021013.

(5) Susi, T.; Dellby, N.; Hayner, R.; Hofer, C.; Kotakoski, J.; Lovejoy, T. C.; Mangler, C.; Mittelberger, A.; Pennycook, T. J.; Plotkin-Swing, B. Open-Source Phase Reconstructions of Focused-Probe 4D-STEM Data with Near-Ideal Direct-Electron Detection. *Microscopy and Microanalysis* **2024**, *30*, ozae044.920.

(6) Hofer, C.; Madsen, J.; Susi, T.; Pennycook, T. J. Detecting charge transfer at defects in 2D materials with electron ptychography. 2023; `https://arxiv.org/abs/2301.04469v4`, URL accessed: 2025-01-06.

(7) Kühne, T. D. et al. CP2K: An electronic structure and molecular dynamics software package - Quickstep: Efficient and accurate electronic structure calculations. *The Journal of Chemical Physics* **2020**, *152*, 194103.

(8) Goedecker, S.; Teter, M.; Hutter, J. Separable dual-space Gaussian pseudopotentials. *Physical Review B* **1996**, *54*, 1703–1710.

(9) Perdew, J. P.; Burke, K.; Ernzerhof, M. Generalized Gradient Approximation Made Simple. *Physical Review Letters* **1996**, *77*, 3865–3868.

(10) Henkelman, G.; Uberuaga, B. P.; Jónsson, H. A climbing image nudged elastic band method for finding saddle points and minimum energy paths. *The Journal of Chemical Physics* **2000**, *113*, 9901–9904.

(11) Madsen, J.; Susi, T. The abTEM code: transmission electron microscopy from first principles. *Open Research Europe* **2021**, *1*, 24.

(12) Mannebach, E. M. et al. Dynamic Structural Response and Deformations of Monolayer MoS_2 Visualized by Femtosecond Electron Diffraction. *Nano Letters* **2015**, *15*, 6889–6895.

(13) Susi, T.; Madsen, J.; Ludacka, U.; Mortensen, J. J.; Pennycook, T. J.; Lee, Z.; Kotakoski, J.; Kaiser, U.; Meyer, J. C. Efficient first principles simulation of electron scattering factors for transmission electron microscopy. *Ultramicroscopy* **2019**, *197*, 16–22.

(14) Trentino, A.; Mizohata, K.; Zagler, G.; Längle, M.; Mustonen, K.; Susi, T.; Kotakoski, J.; Åhlgren, E. H. Two-step implantation of gold into graphene. *2D Materials* **2022**, *9*, 025011.

(15) Li, H.; Wang, S.; Sawada, H.; Han, G. G. D.; Samuels, T.; Allen, C. S.; Kirkland, A. I.; Grossman, J. C.; Warner, J. H. Atomic Structure and Dynamics of Single Platinum Atom Interactions with Monolayer MoS2. *ACS Nano* **2017**, *11*, 3392–3403.

(16) Längle, M.; Mayer, B. M.; Madsen, J.; Propst, D.; Bo, A.; Kofler, C.; Hana, V.; Mangler, C.; Susi, T.; Kotakoski, J. Defect-engineering hexagonal boron nitride us-

ing low-energy Ar+ irradiation. 2024; http://arxiv.org/abs/2404.07166, URL accessed: 2025-01-10.

(17) Komsa, H.-P.; Kurasch, S.; Lehtinen, O.; Kaiser, U.; Krasheninnikov, A. V. From point to extended defects in two-dimensional MoS2: Evolution of atomic structure under electron irradiation. *Physical Review B* **2013**, *88*, 035301.

(18) Peña, F. d. l. et al. hyperspy/hyperspy: v2.2.0. 2024; https://zenodo.org/records/14057415, URL accessed: 2025-01-25.

(19) Campos-Roldán, C. A.; Chattot, R.; Filhol, J.-S.; Guesmi, H.; Pailloux, F.; Bacabe, R.; Blanchard, P.-Y.; Zitolo, A.; Drnec, J.; Jones, D. J.; Cavaliere, S. Structure Dynamics of Carbon-Supported Platinum-Neodymium Nanoalloys during the Oxygen Reduction Reaction. *ACS Catalysis* **2023**, *13*, 7417–7427.
